# Demographic and injury trends for car crash casualties hospitalized in Level I Trauma centers over two decades: data from the National Trauma Registry

**DOI:** 10.1186/s13584-024-00613-z

**Published:** 2024-05-29

**Authors:** Sharon Goldman, Irit Cohen-Manheim, Irina Radomislensky, Bella Savitsky, Israel Trauma Group, H. Bahouth, A. Bar, A. Braslavsky, D. Czeiger, D. Fadeev, A. L. Goldstein, I. Grevtsev, G. Hirschhorn, I. Jeroukhimov, A. Kedar, Y. Klein, A. Korin, B. Levit, I. Schrier, A. D. Schwarz, W. Shomar, D. Soffer, M. Weiss, O. Yaslowitz, I. Zoarets, Moran Bodas

**Affiliations:** 1 Israel National Center for Trauma and Emergency Medicine Research, Gertner Institute for Epidemiology and Health Policy Research , Chaim Sheba Medical Center, 52621, Tel-Hashomer, Ramat Gan, Israel; 2 School of Health Sciences, Ashkelon Academic College, Bella Savitsky, Ashkelon, Israel; 3 Department of Emergnecy & Disaster Management, School of Public Health, Faculty of Medical and Health Sciences, Tel Aviv University, Tel Aviv, Israel

**Keywords:** Trauma, Car occupants, Injury, Trauma registry, Outcomes

## Abstract

**Background:**

During the past two decades, there have been many changes in automotive and medical technologies, road infrastructure, trauma systems, and demographic changes which may have influenced injury outcomes. The aim of this study was to examine injury trends among traffic casualties, specifically private car occupants, hospitalized in Level I Trauma Centers (TC).

**Methods:**

A retrospective cohort study was performed based on data from the Israel National Trauma Registry. The data included occupants of private cars hospitalized in all six Level I TC due to a traffic collision related injury between January 1, 1998 and December 31, 2019. Demographic, injury and hospitalization characteristics and in-hospital mortality were analyzed. Chi-squared (*X*^*2*^) test, multivariable logistic regression models and Spearman’s rank correlation were used to analyze injury data and trends.

**Results:**

During the study period, 21,173 private car occupants (14,078 drivers, 4,527 front passengers, and 2,568 rear passengers) were hospitalized due to a traffic crash. The percentage of females hospitalized due to a car crash increased from 37.7% in 1998 to 53.7% in 2019. Over a twofold increase in hospitalizations among older adult drivers (ages 65+) was observed, from 6.5% in 1998 to 15.7% in 2018 and 12.6% in 2019. While no increase was observed for severe traumatic brain injury, a statistically significant increase in severe abdominal and thoracic injuries was observed among the non-Jewish population along with a constant decrease in in-hospital mortality.

**Conclusions:**

This study provides interesting findings regarding injury and demographic trends among car occupants during the past two decades. Mortality among private car occupant casualties decreased during the study period, however an increase in serious abdominal and thoracic injuries was identified. The results should be used to design and implement policies and interventions for reducing injury and disability among car occupants.

## Background

Road casualties are one of the leading causes of injury, disability and mortality. Globally, road traffic crashes are one of nine leading causes of mortality, with over one million annual deaths, as reported by the World Health Organization [1]. Mortality and injury trends can be attributed to changes in trauma systems, infrastructure, car safety improvements, and road-user behaviors. Both medical professionals and policy makers have been challenged in designing treatments and interventions in an effort to reduce and prevent crash related injuries and disabilities.

In Israel, road traffic casualties account for 25% of all trauma related hospitalizations, but over 40% of severe hospitalized casualties. During the last decade there has been a statistically significant upward trend in the percentage of severe and critically injured from the total hospitalized patients in the trauma centers [2]. Conversely, a substantial decline in mortality has been reported [3–5]. A national report for 2000–2019, illustrated an increase in severely and critically injured hospitalized casualties and a decrease in in-hospital mortality, due to all type traffic related accidents. 2) Specifically, traffic related mortality decreased from 636 deaths in 1998 to 355 deaths in 2019, as reported by the Central Bureau of Statistics (CBS) [6]. According to the CBS, the road traffic fatality rate decreased from 5.6/100,000 residents in 2008 to 3.9/100,000 in 2019 [6, 7]. Similarly, a reduction in traffic related casualties was reported, from 434/100,000 residents in 2008 to 254/100,000 in 2019 [6, 7]. 

While traffic related mortality in Israel has decreased during the past two decades, both the number of cars and the volume traveled has increased tremendously. The number of registered private cars has more than doubled during the last decade. Along with the increase in new vehicles [8], safety systems and accessories have improved dramatically over the past 20 years, including braking systems, airbags, seatbelts, tire pressure systems and many more. For example, while lap seatbelts were the norm, especially in the back seat, today new cars are equipped with a three-point seat belt, which restrains occupants across both their lap and shoulder. The advanced three-point restrain system pulls the seat belt tight and prevents excess slack during a crash, which helps to save lives. Airbags in the front seat were adopted in the mid 1980’s but only in more recent years were they improved for back seat occupants [9]. 

Blind spot detection was developed in response to crashes where the drivers were unable to see the vehicles next to them. Electronic stability was developed to reduce and prevent spin-outs and plow-outs. These systems, which are required in all new cars as of September 1, 2011, use automatic computer-controlled braking of wheels to assist in maintaining control of the vehicle [9]. 

The Israeli CBS reported that 99% of new private cars, in 2019, were installed with at least the majority having only two airbags [10]. In addition, as of 2007, ABS and EPS systems are compulsory in all new vehicles. Other safety systems which have become the norm in all new vehicles include lane departure warning, brake assist and emergency braking systems, and tire pressure six airbags. In comparison, prior to 2001 66% of new cars were installed with airbags, but monitoring systems [11]. Decreasing mortality rates among all trauma patients have been associated with improvements in the Israeli trauma system [3]. Likewise, traffic related mortality has been linked to advances in the Israeli trauma system, including advanced prehospital care (characterized by reduced response time and advanced life support capabilities), specialized trauma units and specific training for trauma care, as well as definitive surgical trauma care courses and more [5]. Trauma registries in general, and specifically The Israel National Trauma Registry (INTR), have proven to be valuable databases, for monitoring and improving quality of trauma care [12]. The INTR, which was established in 1995, monitors the quality of care, measures morbidity and mortality outcomes of trauma patients, and provides evidence based data to policy makers at both the hospital and the national level.

The current study used the INTR database to examine demographic and injury trends among hospitalized road casualties, specifically private car occupants, during the past 22 years. The outcomes provide a glance at transformations which have occurred and will enable policy makers to evaluate past practices and develop future interventions for improving road safety, injury prevention and treatment. The aim of this study was to examine injury trends among hospitalized road casualties, specifically private car occupants.

## Methods

A historical prospective cohort study was performed utilizing data from the INTR for hospitalized patients between January 1, 1998 and December 31, 2019. The INTR includes all hospitalized trauma patients with an ICD-9-CM diagnosis code 800-989.9 who were admitted to the Emergency Department (ED) and hospitalized, died in the ED, or were transferred to or from another hospital. The registry does not include casualties who died at the scene, on the way to the hospital, or following admission 72 h or more after the event. Data in the INTR is anonymous. Additional information regarding the INTR has been previously reported [13]. 

This study received approval from the Sheba Medical Center Institutional Review Board (IRB) (SMC 5138–18). The comprehensive data provided by INTR included hospitalized trauma patients from all six Level I Trauma Centers (TC) in Israel.

Since this research examined injury trends during a 22-year period, it was crucial to include only the TCs whom participated during the entire study period. Thus, for the purposes of this study, only patients hospitalized in a Level I TC were included. The level I TCs are located in all geographical areas throughout Israel and thus represent the entire Israeli population. Inclusion criteria included Israeli residents, ages 16 and older, who were hospitalized due to a traffic injury as occupants of a private car.

### Study variables

- Demographic characteristics: age, gender (males/female), ethnicity (Jew/non-Jew).
- Seat position: drivers (DR), front passenger (FP), rear passenger (RP).
- Year of injury.
- Hospital resource utilization: Intensive Care Unit (ICU), surgical procedure, length of hospital stay (LOS).
- Injury Severity Score (ISS): The sum of the squares of the single highest Abbreviated Injury Scale (AIS) score for each of the three most severely injured body regions categorized 1–8 (mild), 9–14 (moderate), 16–24 (severe), or 25–75 (critical) [14, 15].
- Body regions: based on AIS and includes nine body regions (head, face, neck, thorax, abdomen, spine, upper and lower extremities, and external) [16]. An AIS score *≥* 3 was considered a serious injury. The incidence of serious (AIS *≥* 3) Traumatic Brain Injury (TBI), thoracic and abdominal injuries were evaluated. In addition, aortic injuries (AIS *≥* 3) (Yes/no) were analyzed.
- Ejection from car: yes/no/unknown.

### Statistical analysis

Descriptive data were analyzed using the Chi-squared (χ^2^) test and Spearman’s was used to analyze trends. Logistic regression models were carried out to predict the risk of selected injuries and injury severity, adjusted for gender, race, age, year of hospitalization and occupant seat position. Odds ratios (OR) and 95% confidence intervals (CI) were computed. Odds Ratio were calculated for occupant seat position, age, gender and ethnicity. A value of *p* < 0.05 was considered statistically significant. Statistical analyses were performed using SAS software, version 9.4 (SAS Institute, Cary, NC, USA).

## Results

### Demographic and injury characteristics

During the study period, 21,173 private car occupants were injured and hospitalized in all six Level I TCs Israel; 14,078 DR, 4,527 FP and 2,568 RP, Table 1. A quarter of the drivers were young adults (ages 16–24), 48.4% were between ages 25–44, 17.7% were between 45 and 64 and 9.3% were ages 65+. Age distribution of FP and RP are presented in Table 1. Among DRs, 60% were men while among the passengers 38.3% and 44.3% were males, respectively for FP and RP. The majority of hospitalized drivers were Jewish (75.3%); however, 32.5% of all hospitalized car occupants were non-Jews, while their proportion in population ranges between 21 and 26% during the study period, Table 1 [17]. 

**Table 1:** Demographic, injury and hospitalization characteristics of private car casualties, 1998–2019 (*n* = 21,173)

	Vehicle driver		Front Passenger		Rear passenger		*p*-value^1^
	*n*	%	*n*	%	*n*	%	
Total	**14,078**	***100***	**4,527**	***100***	**2,568**	***100***	
Age							*< 0.0001*
16–24	3,469	*24.6*	1,635	*36.1*	1,235	*48.1*	
25–44	6,807	*48.4*	1,692	*37.4*	751	*29.2*	
45–64	2,494	*17.7*	677	*14.9*	336	*13.1*	
65+	1,308	*9.3*	523	*11.6*	246	*9.6*	
Gender^2^							*< 0.0001*
Females	5,643	*40.1*	2,794	*61.7*	1,430	*55.7*	
Males	8,430	*59.9*	1,733	*38.3*	1,138	*44.3*	
Unknown	5	*0.04*	0		0		
Ethnicity^2^							*< 0.0001*
Jews	10,862	*77.3*	3,278	*72.5*	1,811	*70.6*	
Non-Jews	3,196	*22.7*	1,242	*27.5*	754	*29.4*	
Unknown	20	*0.1*	7	*0.2*	3	*0.1*	
Injury Severity^2^							*< 0.0001*
Minor (ISS 1–8)	8,606	*61.2*	2,634	*58.3*	1,316	*51.4*	
Moderate (ISS 9–14)	2,438	*17.4*	825	*18.3*	600	*23.4*	
Severe (ISS 16–24)	1,386	*9.9*	469	*10.4*	320	*12.5*	
Critical (ISS 25+)	1,622	*11.5*	591	*13.1*	326	*12.7*	
Unknown	26	*0.2*	8	*0.2*	6	*0.2*	
Traffic crash event							
Ejection from car	65	*0.5*	65	*1.5*	87	*3.4*	< 0.0001
Skidding	196	*1.4*	56	*1.2*	37	*1.4*	
Injury Type (AIS *≥* 3)							
TBI	1,615	*11.5*	521	*11.5*	378	*14.7*	< 0.0001
Abdominal	884	*6.3*	385	*8.5*	209	*8.1*	< 0.0001
Thoracic	2,623	*18.6*	965	*21.3*	509	*19.8*	*0.0003*
Aorta	81	*0.6*	40	*0.9*	14	*0.6*	*0.63*
In-hospital Mortality	334	*2.4*	138	*3.1*	62	*2.4*	*0.04*
There of: Severe/critical (ISS16+)	318	*10.6*	131	*12.4*	56	*8.7*	*0.05*
LOS^2^							
0–6 days	10,478	*74.4*	3,291	*72.7*	1,765	*68.7*	*< 0.0001*
7 + days	3,584	*25.5*	1,232	*27.2*	802	*31.2*	
Unknown	16	*0.1*	4	*0.09*	1	*0.04*	
*ICU* ^*2*^							
* (1 + days)*	2,142	*15.2*	743	*16.4*	463	*18.1*	*0.0007*
* Unknown*	6	*0.04*	0	*0*	2	*0.08*	
There of: Severe/critical (ISS16+)	1,754	*58.4*	626	*59.1*	370	*57.5*	0.808

Abbreviations ISS = Injury Severity Score, LOS = Length of Hospital Stay, ICU = Intensive Care Unit, TBI = Traumatic Brain Injury

^1^Chi square is calculated without missing data

^2^Missing data for gender, ethnicity, ISS, LOS and ICU < 0.5%.

While the majority (78.6%) of hospitalizations were due to minor and moderate injuries (ISS 1–14), over 20% were due to severe and critical injuries (ISS 16+). Among drivers, 21.4% suffered from a severe/critical injury, which increased to 23.5% among FP and 25.2% among RP. In-hospital mortality ranged between 2.4 and 3.1% for drivers and passengers, while amongst severe/critical casualties the in-hospital mortality rate was 8.7% among RP, 10.6% among DR and 12.4% among FP (*p* = 0.05), Table 1.

Among severe and critical casualties (ISS 16+) 68% were hospitalized for over one week. Admissions to the ICU was reported for 58% of severe and critical casualties (58.4%, 59.1 and 57.6%, respectively for DR, FP and RP) Table 1. Among those severe and critical patients admitted to the ICU, 72% were males, and 39.4% were young adults, between ages 16–24.

The incidence of serious (AIS *≥* 3) TBI, abdominal and thoracic injuries was analyzed. Traumatic brain injuries were more prevalent among RP (14.7%) in comparison to DR and FP (11.5%; *P* < 0.0001). Almost 20% of hospitalized casualties suffered from a serious thoracic injury (18.6%, 21.3%, and 19.8% respectively for drivers, FP, and RP). Severe abdominal injuries were significantly greater among passengers in comparison to drivers (6.3%, 8.5%, 8.1%, respectively for DR, FP, and RP; *p* < 0.0001). A total of 135 casualties were reported to suffer from either a thoracic aortic or abdominal aortic injury (AIS *≥* 3). There was no statistically significant difference between passenger seat position. (Table 1*)* In- hospital mortality for casualties with aortic injuries was 25.2% (*n* = 34). In comparison, in-hospital mortality among casualties with TBI (AIS *≥* 3) was 13.3%. (*not displayed*)

Multivariable regression analyses, adjusted for age, gender, ethnicity, seat position, and year of hospitalization, were used to calculate the odds of being hospitalized with an injury (Table 2). Males, in comparison to females, were at greater risk for being hospitalized with a severe/critical injury (ISS 16+) (OR = 2.29, 95%CI: 2.13–2.48); serious TBI (OR = 2.32, 95% CI 2.11–2.56) or a serious thoracic injury (OR = 2.11, 95% CI:1.95–2.28). The odds for hospitalization with a severe injury was greater among non-Jews in comparison to Jews (OR = 1.4, 95% CI 1.33–1.55). Similarly, non-Jews were 1.4 times more likely than Jews to suffer from chest injuries (OR = 1.4, 95% CI 1.29–1.53). Front passengers were slightly more likely than drivers to be hospitalized with a severe injury, abdominal or thoracic injury, while RP were more likely to suffer from a severe injury, TBI or abdominal injury. (Table 2)

**Table 2:** Multivariable logistic regression for the association between selected characteristics and injuries among traffic casualties, 1998–2019

Dependent	Model 1		Model II		Model III		Model IV	
Variable	ISS16+		TBI (AIS *≥* 3)		Abdomen (AIS *≥* 3)		Chest (AIS *≥* 3)	
	**OR**	***(95% CI)***	**OR**	***(95% CI)***	**OR**	***(95% CI)***	**OR**	***(95% CI)***
Occupant								
position								
DR	1		1		1		1	
FP	1.29	*(1.11–1.41)*	1.12	*(1.01–1.25)*	1.43	*(1.26–1.63)*	1.35	*(1.24–1.47)*
RP	1.35	*(1.22–1.50)*	1.37	*(1.20–1.55)*	1.31	*(1.11–1.54)*	1.19	*(1.06–1.32)*
Age								
16–24	1		1		1		1	
25–44	0.74	*(0.69–0.81)*	0.64	*(0.57–0.71)*	0.69	*(0.61–0.78)*	0.78	*(0.71–0.85)*
45–64	1.06	*(0.96–1.14)*	0.8	*(0.71–0.91)*	0.74	*(0.63–0.87)*	1.26	*(1.14–1.40)*
65+	1.21	*(1.07–1.36)*	1.19	*(1.03–1.37)*	0.62	*(0.50–0.77)*	1.5	*(1.35–1.71)*
Gender								
Female	1		1		1		1	
Male	2.29	*(2.13–2.48)*	2.32	*(2.11–2.56)*	1.55	*(1.38–1.75)*	2.11	*(1.95–2.28)*
Ethnicity								
Jew	1		1		1		1	
Non-Jew	1.4	*(1.33–1.55)*	1.32	*(1.19–1.46)*	1.26	*(1.11–1.42)*	1.41	*(1.29–1.53)*

Abbreviations FP = front passenger, RP = rear passenger, DR = driver, ISS = Injury Severity Score, TBI = Traumatic Brain Injury, OR = Odds Ratio, CI = Confidence Interval, AIS = Abbreviated Injury Scale

Multivariable model was adjusted for year of hospitalization, gender, ethnicity, seat position and injury severity

### Trends

**Fig. 1:**
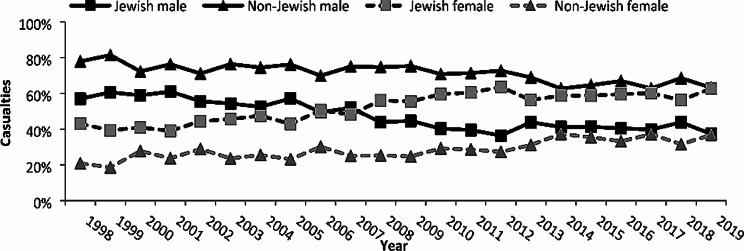
Gender and ethnicity injury trends among private car occupant casualties,1998–2019. Hospitalization trends due to a private car traffic injury were observed for Jews and non-Jews. An inverse trend was observed for both Jews and non-Jews, the percentage of hospitalized females increase steadily from 1998 to 2019 (*p* < 0.0001)

Trends for demographic, injury and hospitalization characteristics were examined and significant changes in the distribution of gender, age and ethnicity were observed. The percentage of females hospitalized as occupants in a private car increased from 37.7% in 1998 to 53.7% in 2019 (Fig. 1). Jewish females comprised 43% of the hospitalizations in 1998, which increased to 62.7% in 2019; in comparison, non-Jewish females comprised only 21% of the hospitalizations in 1998, which increased to 36.8% in 2019 (*p* < 0.0001). Similarly, the percentage of female drivers (both Jewish and non-Jewish), increased significantly during the study period. In 1998, 33% of the hospitalized Jewish drivers were females, which increased to 58.65 in 2019 (*p* < 0.0001). Among non-Jews, only 8% of the hospitalized drivers in 1998 were females, which increased to 29.3% in 2019 (*p* < 0.0001).

**Fig. 2:**
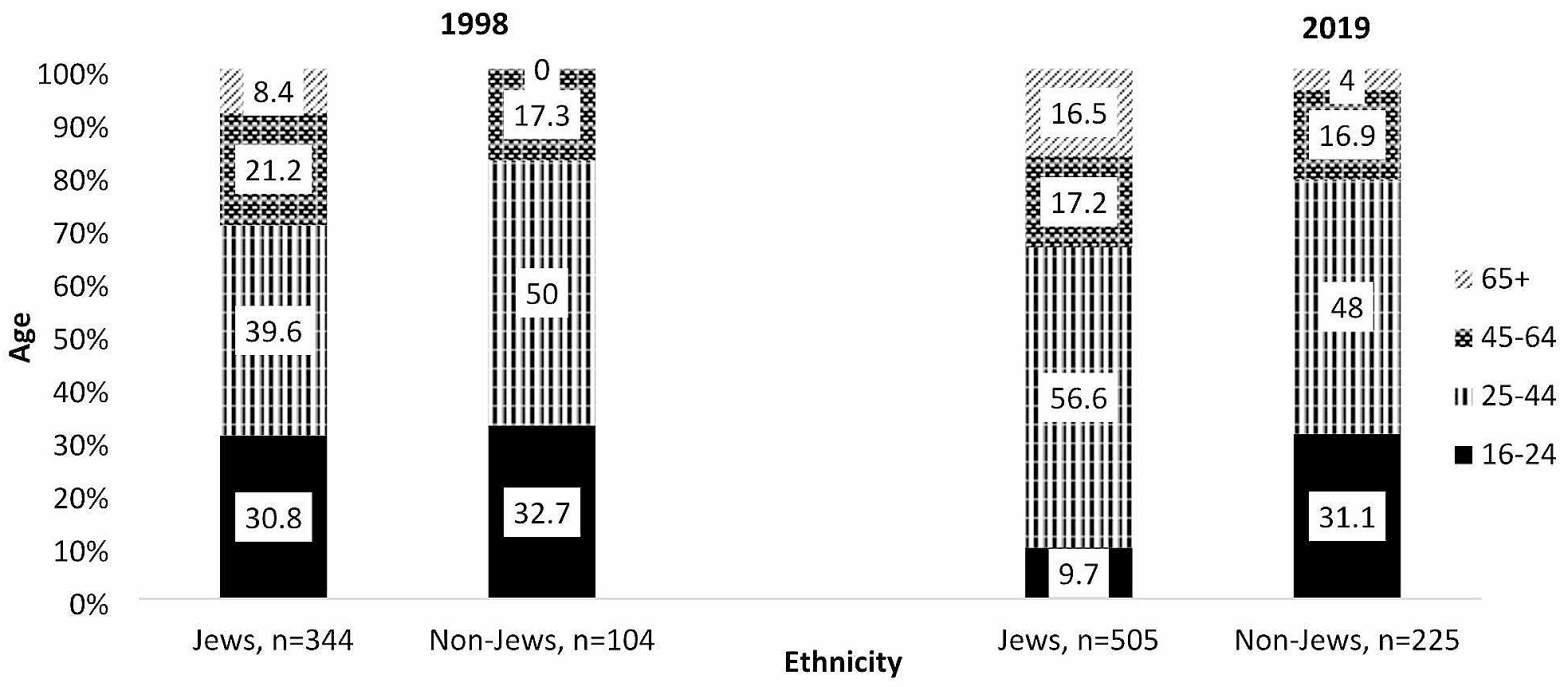
Age distribution of hospitalized drivers by ethnicity, 1998 vs. 2019. Between 1998 to 2019, a dramatic increase in the percentage of hospitalized older drivers (ages 65+) was observed, notably among Jewish drivers; from 8.4% of hospitalized drivers in 1998 to 16.5% in 2019. A smaller increase was noted among non-Jews, from 0 to 4%. *P* < 0.0001

In 1998, older adults (ages 65+) accounted for 6.5% of the drivers, which increased to 12.6% in 2019. While age distribution among non-Jews remained fairly stable over time, among Jews signifant changes were observed. The percentage of young drivers [16–24] decreased from 30.8% in 1998 to 9.7% in 2019, and controversely, the percentage of older drivers (65+) increased from 8.4 to 16.5% in 2019, (Fig. 2).

**Fig. 3:**
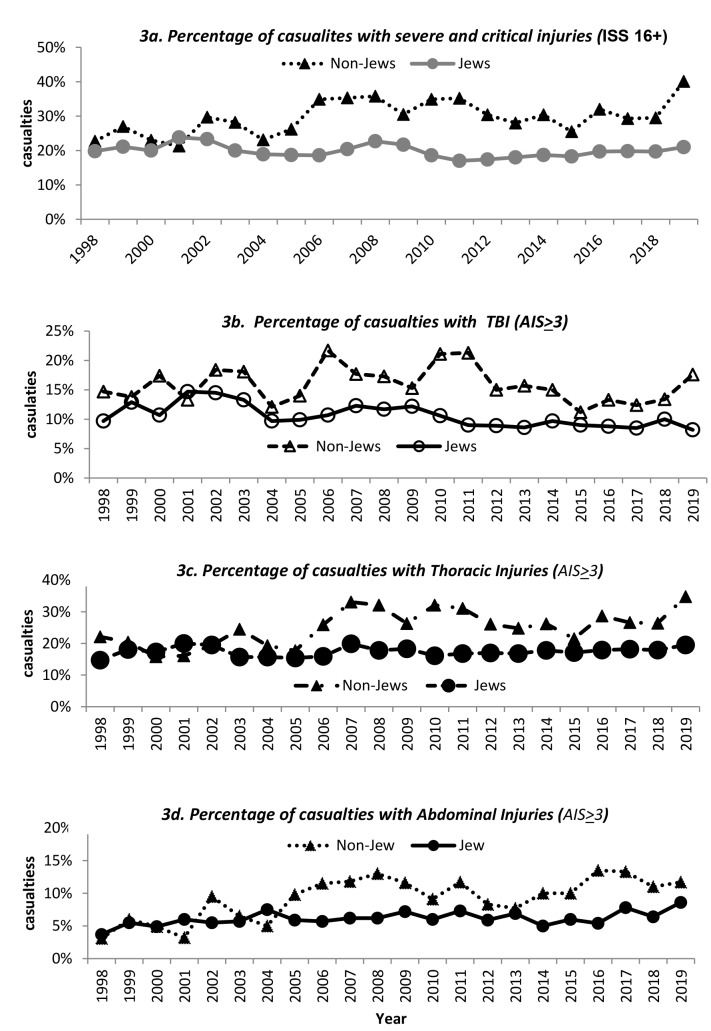
**a**–**d** Selected injury trends by ethnicity and year, 1998–2019. *Spearman’s correlation* was used to analyze the trends between 1998–2019, for Jews and non-Jews. TBI = Traumatic Brain Injury; ISS = Injury Severity Score; AIS = Abbreviated Injury Score. **a** presents the differences between hospitalized Jewish and non-Jewish casualties suffering from severe and critical injuries (ISS 16+). A statistically significant increase was observed for non-Jews hospitalized with a severe/critical injury. *Spearman’s correlation*: rho: Jews = **-**0.396 (*p*=-0.07); non-Jews = 0.498 (*p* = 0.02). Figure **b**. Illustrates the trends of hospitalized Jewish and non-Jewish casualites diagnosed with a severe TBI (AIS *≥* 3*)*. Significant differences in trends were observed between Jews and non-Jews; while Jews showed a significant decrease in hospitalizations, the percentage of non-Jews with TBI varied during the study period. *Spearman’s correlation*: rho Jews= -0.703 (*p* < 0.001), non-Jews =-0.154 (*p* = 0.49). Figure **c**. Illustrates trends for hospitalization due to severe ***(****AIS* *≥* *3****)*** thoracic injuries. A statistically significant increase was observed for non-Jews hospitalized with thoracic injuries (AIS *≥* 3). *Spearman’s correlation*: rho Jews = 0.216 (*p* = 0.33); non-Jews = 0.653 (*p* < 0.001). Figure **d**. Presents injury trends for casualties hospitalized with serious (AIS *≥* 3) abdominal injuries. A significant increase in serious abdominal injuries (AIS *≥* 3) was reported, which was more pronounced among non-Jewish casualties. *Spearman’s correlation*: rho: Jews = 0.512 (*p* = 0.01); non-Jews = 0.685 (*p* < 0.001). For the selected severe injuries, a noticeable increase in hospitalizations among non-Jewish casualties was observed, whereas among Jews the direction of the trend depended on the injury type

Hospitalizations due to a severe or critical (ISS 16+) injury significantly increased among non-Jews, from 22.7% in 1998 to 40.1% in 2019 (*p* = 0.02), while among Jews no significant changes were reported (Fig. 3a.). Significant differences were observed between Jews and non-Jews diagnosed with TBI. While the percentage of Jewish casualties suffering from severe TBI ranged from 9.7% in 1998 to 8.2% in 2019, with a peak of 14.7% in 2001; among non-Jews the percentage increased from 14.7% in 1998 to 17.6% in 2019, with peaks in 2006 (21.7%), 2010 (21.1% and 2011 (21.3%). Figure 3b.

A statistically significant increase was observed for both serious abdominal and thoracic injuries (AIS *≥* 3), see Fig. 3c–d. The percentage of casualties suffering from a serious thoracic injury (AIS *≥* 3) increased from 16.4% in 1998 to 24.8% in 2019, however, the greatest increase was among non-Jews, in which thoracic injuries increased from 22.1% in 1998 to 34.8% in 2019 ( *p* < 0.001), Fig. 3c.

A significant increase in serious abdominal injuries (AIS *≥* 3) was reported; from 3.6% in 1998 to 9.7% in 2019. However, among non-Jews the increase was more pronounced (3.1% in 1998 and 11.7% in 2019, *p* < 0.001), Fig. 3d.

In-hospital mortality among severe/critical casualties decreased from 11.6% in 1998 to 7.4% in 2019 (*p* < 0.01). For each occupant position, a decrease in mortality was observed. It should be noted that the number of in-hospital annual deaths for private car occupants remained low, approximately 20 deaths per year.

### Hospitalization resource utilization

While an increase in severe casualties was observed during the past 22 years, the length of hospital stay (LOS) and admissions to the ICU decreased among these severe and critical patients. Among severe and critical patients (ISS 16+), 60.9% of patients were admitted to the ICU in 1998 compared to 50.2% in 2019. During the entire study period, males with severe and critical injuries were much more likely than females to be admitted to the ICU and for a longer period; 60.9% of males and 52.8% of females were admitted to the ICU; 20% of males and 15% females were hospitalized in the ICU for over 14 days (*P* < 0.0001). A decrease in hospital LOS was observed for all severe and critical casualties. In 1998, 76.8% of the severe/critical casualties were hospitalized for more than one week, which decreased to 61.4% in 2019 (data not shown).

## Discussion

Trauma systems in general, and specifically trauma registries, have shown to be fundamental in improving quality of care and determining injury outcomes in among severe and critical traffic casualties [2, 3, 5, 12]. 

Motor vehicle accidents have a substantial impact on our lives, both from financial, medical and community perspective. The United States Centers for Disease Control and Prevention report that for every one person killed in a motor vehicle accident, another eight are injured and 100 people visit the ED [19]. Seat position has been associated with both injury and mortality. Studies have shown that FP are at greater risk of mortality [18–21], while RP are at greater risk of head and abdominal trauma [20]. In contrast, a recent study by the Insurance Institute for Highway Safety (IIHS) found that RP are at greatest risk for mortality and for injury [21]. 

In Israel, during the last two decades, in-hospital mortality has declined significantly for all private car occupants. In the current study, the risk of mortality was greater among FP in comparison to DR and RP. Decreasing trends in trauma related mortality, and specifically traffic related mortality, have been reported not only in Israel, but also globally [3, 5, 22, 23]. A study of road traffic injuries found that during two decades, 1995–2015, there was a significant reduction in mortality rates both globally and specifically in the Eastern Mediterranean Region. The study concluded that severe injuries decreased by more than half for high and middle income countries, while among low income countries the rates remained high [24]. In the United States, in 2019, a reduction in traffic related death rates was observed, after a five year increase.

While seatbelts have proven to save lives, they are often the cause of contusions across the chest wall and abdominal injuries among restrained car occupants. The seatbelt syndrome, which describes the presence of the seat belt sign plus an intra-abdominal or spinal injury, is present in as many as 30% of road traffic crash casualties admitted to emergency departments. Seatbelt injuries are also affected by gender. In a recent study of gender and seatbelt injuries, females and males suffered from different injuries, with the abdomen being the most prevalent injured site among females (62.9% vs. 27.0%, *p* < 0.0001)and the thorax the most prevalent among males (47.0% vs. 23.5%, *p* < 0.0001) [25]. 

Interestingly, while car safety measures have improved over the past two decades [8, 9], the finding from this study show no reduction in head injuries and a continual increase in the percentage of serious abdominal and thoracic injuries, especially among non-Jews. Possible explanations for the significant increase in thoracic and abdominal injuries may be due to driver behavior; such as, high speed crash impact (resulting in more belt-related pressure on abdomen and thorax) [23]. Socioeconomic and infrastructure inequalities may also play a role, as these population groups may not have newer cars with improved safety accessories. Disparities between Jews and non-Jews could also be contributed to infrastructure. The majority of the Jewish population in Israel lives in urban areas in central Israel, while the non-Jewish population often lives in rural regions in Northern and Southern Israel. That said, many roads in Northern Israel, lack proper infrastructure, such as lane dividers which can prevent high impact collisions [23]. In addition, the socioeconomic position of the Jewish population, in general, is higher than the non-Jewish population, which has been shown to be correlated to injury [26]. Tiruneh also reported differences between Jews and Arabs regarding in-hospital mortality, injury severity, evacuation methods and hospital resource utilization among all hospitalized traffic related casualties [27]. Possible causes contributing to ethnic differences of road accidents in Israel included lower investment in infrastructure, poor traffic enforcement in Arab villages, defiance of state authorities and low socio-economic status, which were perceived as factors contributing to unsafe driving and increased traffic crashes among the Israeli Arab population [28]. 

This study showed a substantial increase in the proportion of hospitalized older adult drivers during the past two decades, which can be associated with an increase in life expectancy, better quality of life and cultural changes. Not only has life expectancy increased, but also the number of older drivers. In 2010 there were 80,000 persons ages 75 + licensed to drive compared to over 153,000 in 2018 [28, 29]. While in the general population female drivers are the norm, (in 2018, women comprise 44% of those with a driver’s license) among Arabs and ultra-religious Jews women drivers are less common [29]. For example, only 32% of ultra-religious women hold a driver’s license, compared to 74% among secular Jewish women(30–31) The proportion of Arab women with a drivers license also increased, from 33% in 2010 to 39% in 2019 [29–31]. 

## Policy suggestions and recommendations

This study used a national trauma registry to focus on traffic injuries among private car occupants. The registry provided data showing both injury and demographic changes during two decades. These outcomes are crucial for both policy makers and medical professionals in developing updated programs and protocols. Intervention programs should be culturally adapted with a focus on elderly drivers and the non-Jewish population. For example, programs targeting elderly drivers might include (1) mandatory regular testing for vision tests, reaction time assessment and cognitive evaluation; (2) alternative transportation options including ridesharing services, community transportation or shuttle services for elderly to essential destinations like medical appointments and grocery stores; and (3) financial incentives for transitioning from driving to using alternative transportation options.

Interventions focusing on the non-Jewish community could include incentives for safe driving such as insurance discounts and reduced license fees as well as improving road infrastructure. Such infrastructure improvements in non-Jewish communities might include better lighting on the roads, signage and traffic management systems. Emergency room (ER) staff should be made aware of the increase in thoracic injuries among traffic casualties, and improve their ability to effectively identify and manage thoracic injuries. Training for ER staff should include identification of signs and symptoms, diagnostic techniques and treatment options. ER staff should be encouraged to maintain a high index of suspicion for thoracic trauma among traffic casualties, especially in patients with related symptoms and they should be encouraged to use prompt diagnostic imaging to assess potential thoracic injuries. Early recognition will enable more appropriate treatment leading to better outcomes. Trauma registries should continue to be used to identify at risk population groups and to design recommendations for reducing morbidity and mortality among car occupants.

### Limitations

This study is based on data from the INTR, which includes only hospitalized casualties. Thus, fatalities at the scene of the accident or on the way to the hospital were not included. However, since the trauma registry includes all Level I trauma centers in Israel, the majority of severe and critical patients receiving definitive care were accounted for. It should be noted, that data from the INTR show that among severe and critical casualties (ISS 16+), 74% were hospitalized in Level 1 TCs and 26% in Level II centers.

This study did not examine the efficacy of motor vehicle safety devices or trauma systems, but rather took a look at changes of injury trends and casualty characteristics during the past two decades. The correlation between risky behaviors, unsafe road practices and the increased risk of severe and critical injuries has been previously reported and thus future research should focus on examining the causes leading to changes in injury trends. For this study, educated theories suggest that behavioral changes, improved technologies, and regulations are associated with injury trends. .

## Conclusion

The current study used the INTR database to examine demographic and injury trends among private car traffic casualties hospitalized in Level I Trauma Centers during 22 years. While mortality among private car occupant casualties decreased during the study period, an increase in serious abdominal and thoracic injuries was identified. Upgraded car safety features along with improved trauma systems shape the injuries and outcomes from traffic accidents in general, and specifically among car occupants. The findings provide policy makers with evidence based data to design culturally appropriate interventions and policies for reducing injury and disability among car occupants, as well as confirm the importance of a national trauma registry and a highly developed trauma system.

## Data Availability

Not applicable.

## Abbreviations

INTR: Israel National Trauma Registry
IRB: Institutional Review Board
SMC: Sheba Medical Center
TC: Trauma center
DR: Driver
FP: Front passenger
RP: Rear passenger
ISS: Injury Severity Score
AIS: Abbreviated Injury Score
ED: Emergency Department
ICU: Intensive Care Unit
LOS: Length of stay
TBI: Traumatic Brain Injury
OR: Odds Ratio
CI: Confidence interval
CBS: Central Bureau of Statistics
IIHS: Insurance Institute for Highway Safety
NHTSA: National Highway Traffic Safety Administration
